# Epigenetic Regulation of Mitochondrial Quality Control Genes in Multiple Myeloma: A Sequenom MassARRAY Pilot Investigation on HMCLs

**DOI:** 10.3390/jcm10061295

**Published:** 2021-03-21

**Authors:** Patrizia D’Aquila, Domenica Ronchetti, Maria Eugenia Gallo Cantafio, Katia Todoerti, Elisa Taiana, Fernanda Fabiani, Alberto Montesanto, Antonino Neri, Giuseppe Passarino, Giuseppe Viglietto, Dina Bellizzi, Nicola Amodio

**Affiliations:** 1 Department of Cell Biology, Ecology and Earth Sciences, University of Calabria, 87036 Rende, Italy; d_patrizia2002@yahoo.it (P.D.); alberto.montesanto@unical.it (A.M.); giuseppe.passarino@unical.it (G.P.); dina.bellizzi@unical.it (D.B.); 2 Department of Oncology and Hemato-Oncology, University of Milan, 20122 Milan, Italy; domenica.ronchetti@unimi.it (D.R.); katiatodoerti@gmail.com (K.T.); elisa.taiana@unimi.it (E.T.); antonino.neri@unimi.it (A.N.); 3 Hematology, Fondazione Cà Granda IRCCS Policlinico, 20122 Milan, Italy; 4 Department of Experimental and Clinical Medicine, Magna Graecia University of Catanzaro, 88100 Catanzaro, Italy; eugy.2186@gmail.com (M.E.G.C.); viglietto@unicz.it (G.V.); 5 Medical Genetics, University “Magna Graecia”, 88100 Catanzaro, Italy; fernandafabiani@libero.it

**Keywords:** cancer epigenetics, methylation, Sequenom MassARRAY

## Abstract

The mitochondrial quality control network includes several epigenetically-regulated genes involved in mitochondrial dynamics, mitophagy, and mitochondrial biogenesis under physiologic conditions. Dysregulated expression of such genes has been reported in various disease contexts, including cancer. However, their expression pattern and the possible underlying epigenetic modifications remain to be defined within plasma cell (PC) dyscrasias. Herein, we compared the mRNA expression of mitochondrial quality control genes from multiple myeloma, plasma cell leukemia patients and human myeloma cell lines (HMCLs) with healthy plasma cells; moreover, by applying the Sequenom MassARRAY EpiTYPER technology, we performed a pilot investigation of their CpG methylation status in HMCLs. Overall, the results provided indicate dysregulated expression of several mitochondrial network’s genes, and alteration of the CpG methylation profile, underscoring novel potential myeloma biomarkers deserving in-depth functional investigation in the future.

## 1. Introduction

Multiple myeloma (MM) is a complex haematological disease mostly occurring in older adults, in which plasma cells (PCs) undergo malignant transformation through a stepwise process evolving from premalignant conditions, such as monoclonal gammopathy of undetermined significance (MGUS) and smouldering multiple myeloma (SMM), to overt MM [1,2,3,4]. A plethora of chromosomal abnormalities and mutational events, affecting oncogenes, tumor suppressor genes and cell cycle regulators, contributes to the heterogeneous landscape of MM and ultimately leads to dysregulation of signalling pathways, prompting myelomagenesis [5,6,7,8]; in parallel, deregulated epigenetic mechanisms, such as aberrant DNA methylation and histone modifications, as well as deranged microRNA (miRNA) networks, have been found implicated in the onset and progression of MM, and more specifically in the clonal heterogeneity and response to treatment [9,10,11,12,13,14,15,16,17,18].

Accumulating evidence has recently demonstrated that mitochondria-driven metabolic reprogramming is involved in cancer development and progression [19,20]. Mutations in both coding and non-coding regions of mitochondrial DNA (mtDNA) have been reported in various cancer types, such as colorectal, lung, prostate, breast, and gastric carcinomas [21,22,23,24]; moreover, alterations in the mitochondrial quality control system, which include pathways coordinating biogenesis and mitophagy as well as mitochondrial fusion and fission processes, unbalance the mitochondrial homeostasis and function, contributing to the pathogenesis and progression of various cancers [25,26,27,28,29].

Consistently, a fragmented mitochondrial network, along with elevated fission and/or decreased fusion activities, has been associated with a migratory phenotype in several cancers [30,31]; mitophagy is also emerging as both a positive and negative regulator of tumorigenesis, depending on the context and the cancer stage [32].

In MM cells, abnormal expression of genes encoding mitochondrial anti-oxidants, Ca^2+^ channels, and anti-apoptotic protein, and their association with bortezomib resistance, has been reported [33]; moreover, Zhan et al. reported an iron-dependent expression of several mitochondrial biogenesis-related genes in MM patients’ PCs, which associated with disease progression and inferior clinical outcome [34]. An epigenetic control of genes coding for components of the mitochondrial quality control system has emerged in both pathological and physiological conditions, including cell development, aging, hypertension, and diabetic retinopathy, but very little is known about their expression pattern and the underlying regulatory mechanisms in PC dyscrasias [35,36,37,38].

To this purpose, we here investigated the transcription pattern and performed a focused methylation analysis, by applying the Sequenom MassARRAY EpiTYPER technology, of the CpG islands located within candidate genes involved in mitochondrial biogenesis, mitophagy, fusion, and fission to preliminarily address the epigenetic variability of these sites in MM PCs.

## 2. Experimental Section

### 2.1. DNA Samples

MM1s and KMS11 human multiple myeloma cell lines (HMCLs) were obtained from the Leibniz Institute DSMZ (German Collection of Microorganisms and cell cultures GmbH) and the Japanese Collection of Research Bioresources (National Institute of Health Sciences Japan), respectively. HMCLs were grown in RPMI1640 medium supplemented with 10% FBS and 1% P/S, as previously reported [39]. Ten healthy human samples with an average age of 63.1 years (range: 50–69) were analysed as control samples. Fully informed consent was obtained in writing from all the participants, and all the studies were approved by the “Regione Calabria Ethics Committee, section Area Nord” (Prot. CE 119/2016). Six millilitres of venous blood were drawn from each human subject. Plasma/sera were used for routine laboratory analyses, while DNA was extracted from buffy coats following standard procedures. Genomic DNA was obtained by phenol/chloroform purification; DNA concentration and purity were determined spectrophotometrically.

### 2.2. Gene Expression Profiling (GEP)

Total RNA samples from fifty newly diagnosed MM samples (median age: 67; range: 41–78), 15 primary plasma cell leukemia (PCL; median age: 59; range: 48–79), 4 normal controls (median age: 23; range: 21–24; purchased from Voden, Medical Instruments IT) and 10 HMCLs were profiled onto GeneChip^®^ Human Gene 2.0 ST arrays (Thermo Scientific, Wilmington, DE, USA) according to the manufacturer’s procedure as previously described [40]. Data processing and normalization was performed by a Robust Multi Array Average (RMA) algorithm. A custom annotation pipeline that comprises GENCODE v25 (Ensembl v87) annotations and CDF (Chip Definition File) version 21 for gene annotations freely available at http://brainarray.mbni.med.umich.edu/Brainarray/Database/CustomCDF/21.0.0/genecodeg.asp has been adopted in order to exclude ambiguous, unspecific probes. All the data have been deposited in the NCBI Gene Expression Omnibus database (GEO; http://www.ncbi.nlm.nih.gov/geo) and are accessible under accession #GSE 116294.

### 2.3. Primer Design for EpiTYPER Assay

PCR primers for the methylation study were designed using Sequenom’s EpiDesigner software using the following precautions: absence of CpGs, concurrent amplification of both methylated and unmethylated templates, amplicons less than 300 bp in length, maximum CpG coverage. Forward and reverse primer sequences were reported elsewhere [35].

### 2.4. Bisulfite Treatment and PCR Conditions

Bisulfite treatment of DNA samples was performed using the EZ-96 DNA Methylation-Gold kit (Zymo Research, Euroclone, Milan, Italy), according to the manufacturer’s protocol. Briefly, 1 μg of genomic DNA was mixed to 130 μL of CT conversion reagent and incubated at 98 °C for 10 min and, successively, at 64 °C for 2.5 h. After loading 400 μL of M-binding buffer, each sample was loaded on the wells of the silicon-A binding plate and centrifuged at 3000 g for 5 min. Subsequently, samples were rinsed with 400 μL of M-wash buffer and centrifugated at 3000 *g* for 5 min. Then, an incubation for 20 min at room temperature occurred in the presence of 200 μL of M-desulfonation buffer. After a centrifugation at 3000 g for 5 min, wells underwent two consecutive washes with 400 μL of M-wash buffer. Converted DNA was eluted in 30 μL of M-elution buffer. PCR reactions were carried out in a total volume of 5 μL using EpiTaq PCR buffer 1X, 2.5 mM of MgCl_2_, 0.3 mM dNTP mixture, 0.4 μM of each primer, 0.005 U TaKaRa EpiTaq HS (TaKaRa, Diatech Lab Line), and 1 μL of bisulfite-treated DNA. The thermocycling protocol started from a heat activation of the enzyme at 95 °C for 4 min, then pre-degeneration at 94 °C for 20 s, followed by 45 cycles of denaturation at 94 °C for 20 s, annealing at optimal temperature for each primer pair [35] for 30 s, extension at 72 °C for 1 min, then one cycle at 72 °C for 3 min. Successful and specificity of each amplification reaction was ascertained by agarose gel electrophoresis.

### 2.5. Dephosphorylation of Unincorporated Deoxynucleoside Triphosphates, In Vitro Transcription and RNaseA Cleavage

Unincorporated dNTPs were dephosphorylated by adding 1.7 μL DNase free water and 0.3 μL (0.5 U) shrimp alkaline phosphatase (SAP) (Sequenom, Inc., San Diego, CA, USA) and incubating at 37 °C for 40 min; SAP was then heat-inactivated at 85 °C for 5 min. Subsequently, samples were incubated at 37 °C for 3 h with 5 μL of T-cleavage reaction mix (Sequenom), containing 3.21 μL RNAse-free water, 0.89 μL 5X T7 polymerase buffer, 0.22 μL T-cleavage mix, 0.22 μL 100 mM DTT, 0.40 μL T7 RNA polymerase and 0.06 μL RNase A, for concurrent in vitro transcription and base-specific cleavage. The samples of cleaved fragments were then diluted with 20 μL water. The cleavage reaction was conditioned by adding 6 mg of clean resin.

### 2.6. Mass Spectrometry

Ten nl of the cleavage reactions were spotted onto silicon matrix preloaded chips (Spectro-CHIP; Sequenom, Inc., San Diego, CA, USA) by a MassARRAY nanodispenser (Sequenom, Inc., San Diego, CA, USA) and analysed using the MassARRAY Compact System matrix-assisted laser desorption/ionization-time-of-flight mass spectrometer (MALDITOF) (Sequenom, Inc., San Diego, CA, USA). The spectra’s methylation ratios for each CpG units were calculated using EPITYPER software v1.0 (Sequenom, Inc., San Diego, CA, USA). Triplicate analyses from independent sodium bisulfite-treated DNA samples were undertaken. The effectiveness of the entire experimental procedure was ascertained by analysing samples obtained by mixing fully methylated and non-methylated gDNA standards (CpGenome Universal Unmethylated DNA (Chemicon) and CpGenome Universal Methylated DNA, Chemicon, Millipore) with 10% methylation increments. Data quality control and filtering were carried out to exclude the CpG units displaying a success rate lower than 90%.

### 2.7. Statistical Analyses

Descriptive statistics for continuous and categorical variables were used to describe the characteristics of the analyzed samples. Logistic regression analyses were performed to compare the methylation pattern of genes involved in mitochondrial quality control between HMCLs (MM1s and KMS11) with respect to the methylation profiles of healthy control subjects. Statistical analyses were performed using the R statistical language program (http://www.Rproject.org/) using the *CpGassoc* package [41]; a significance level of 0.05 was chosen. For differential analysis of gene expression, Dunn’s test was applied for multiple comparisons between PC dyscrasias’ groups using the R software, with a significance of 0.01.

## 3. Results

### 3.1. Dysregulated Expression of Mitochondrial Quality Control Genes in MM and Its More Advanced Stages

**Figure 1:**
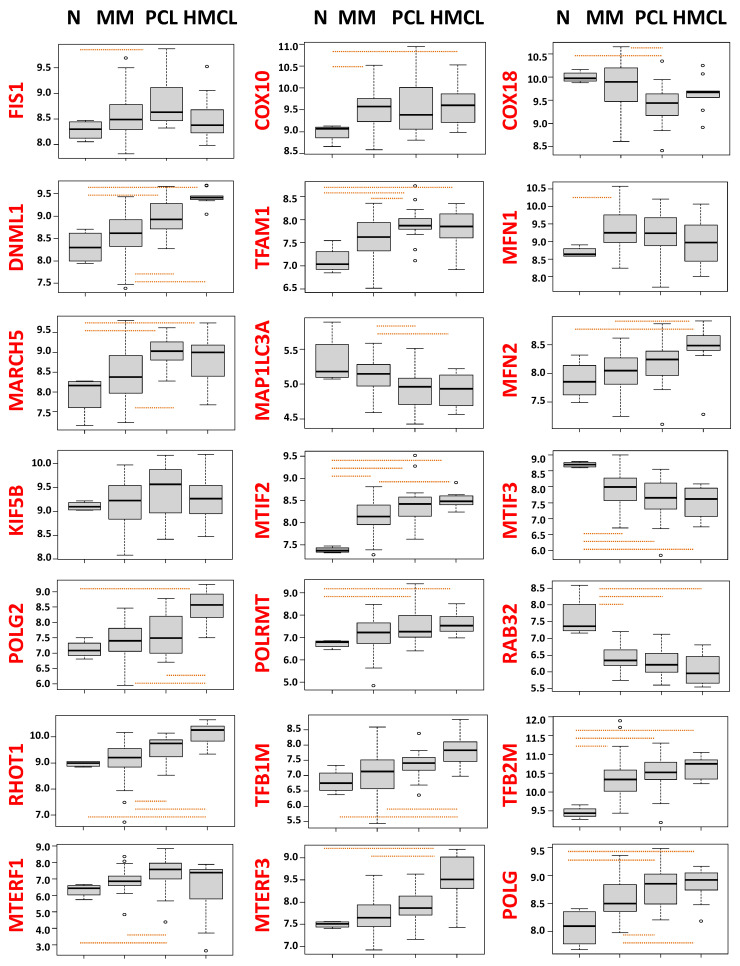
Box plot representation of the mRNA expression of mitochondrial quality control genes, in four normal control (N), 50 multiple myeloma (MM) patients, 15 primary plasma cell leukemia (PCL), and 10 human myeloma cell lines (HMCLs) evaluated by GeneChip^®^ Human Gene 2.0 ST array (GSE 116294). In each panel, orange dashed lines indicate significant differences between groups based on Dunn’s test (*p*-value < 0.01).

We first sought to evaluate, in the context of MM and its more advanced stages, the expression pattern of the most relevant genes involved in mitochondrial quality control systems. To this purpose, we analysed a proprietary gene expression profile dataset (GSE 116294) including MM, PCL patients, HMCLs, as well as healthy PCs. Interestingly, we observed dysregulated expression of most of the mitochondrial quality control genes across the different disease stages. In detail, the expression of *COX10*, *DNM1L*, *FIS1*, *KIF5B*, *MARCH5*, *MFN1*, *MFN2*, *MTERF1*, *MTERF3 MTIF2*, *POLG*, *POLG2*, *POLRMT*, *RHOT1*, *TFAM*, *TFB1M*, and *TFB2M* steadily increased, while the expression of *COX18*, *MAP1LC3A*, *MTIF3*, and *RAB32* transcripts progressively declined from normal donor plasma cells to PCLs. For these genes, dysregulated expression respect to normal cells was also found in HMCLs, with a pattern generally resembling that of PCLs, as expected (Figure 1).

### 3.2. CpG methylation Analysis of Mitochondrial Quality Control Genes

Recent studies have demonstrated that epigenetic pattern of genes involved in mitochondrial quality control are associated with complex phenotypes, including aging, or can be modulated by environmental factors [37,39]. Having found dysregulated expression levels of different genes involved in mitochondrial functions in primary MM samples and HMCLs (Figure 1), and searching for potential mechanisms responsible of the altered expression in tumour cells, we investigated epigenetic marks associated with the promoters of these genes. Taking into account that CpG methylation impacts the expression of these genes in various physiopathologic contexts [35,37], the methylation levels of 838 CpG sites falling in the CpG islands located within the promoter region of *COX10*, *COX18*, *FIS1*, *DNM1L*, *KIF5B*, *MAP1LC3A*, *MARCH5*, *MFN1*, *MFN2*, *MTERF1*, *MTERF3*, *MTIF2*, *MTIF3*, *POLG*, *POLG2*, *POLRMT*, *RAB32*, *RHOT1*, *TFAM*, *TFB1M*, and *TFB2M* genes were evaluated on bisulfite-treated DNA samples using as experimental in vitro models two HMCLs (KMS11 and MM1s) along with healthy control cells, which underwent Sequenom MassARRAY quantitative analysis.

Following quality control criteria (see Experimental Section), the final dataset included 588 CpG sites organized in single sites or CpG units. Such analysis, in which DNA methylation levels are reported as an arithmetic mean of the CpG sites of each gene, revealed that in HMCLs, 15 out of 21 genes showed differences in methylation levels with respect to the healthy control samples (Table 1). In detail, *KIF5B*, *MAP1LC3A*, *MARCH5*, *MFN1*, *MFN2*, *MTERF1*, *MTERF3*, *MTIF3*, *POLG2*, *POLRMT*, *RAB32*, *RHOT1*, *TFAM*, and *TFB1M* were found hypermethylated in at least one HMCL, while just *COX10* gene displayed hypomethylation with respect to the control samples; conversely, *KIF5B*, *MAP1LC3A*, *MARCH5*, *MFN1*, *MTERF1*, *MTERF3*, *MTIF3*, *POLG2*, *POLRMT*, and *RAB32* were hypermethylated, while only *COX10* gene was hypomethylated in both HMCLs. The association results of each CpG unit methylation between HMCLs and healthy control samples are reported in Supplementary Table S1.

## 4. Discussion

Mitochondrial quality control, a process that finely regulates the maintenance of mitochondrial integrity and function, is regulated under physiological conditions and often deregulated in diseases. By sustaining proliferation, apoptosis evasion and drug resistance, such a process has been found implicated in several cancer hallmarks, ultimately fine-tuning bio-energetic and biosynthetic demands of cells to fuel tumour initiation and progression [20]. How these genes are expressed, and whether they are also regulated at the epigenetic level in PC dyscrasias, has not been previously addressed. To this purpose, using a proprietary GEP dataset, we first investigated the expression levels of candidate genes involved in mitochondrial biogenesis, fusion, and fission in RNA samples from MM, PCL patients, HMCLs, as well as healthy PCs. Intriguingly, the results we obtained revealed an extensive remodelling of gene expression that affected more than half of the genes under examination. In detail, mRNA hyperexpression of several genes involved in mitochondrial biogenesis (*MTERF1*, *MTERF3 MTIF2*, *POLG*, *POLG2*, *POLRMT*, *TFAM*, *TFB1M*, *and TFB2M*), fusion/fission (*COX10*, *DNM1L*, *FIS1*, *KIF5B*, *MARCH5*, *MFN1*, and *MFN2*) and mitophagy (*RHOT1*) processes was observed in pathological samples as compared to control cells. Furthermore, a decrease in the expression levels of *COX18* and *MTIF3* genes was detected, which are involved in mitochondrial biogenesis, and *RAB32* and *MAP1LC3A* genes, implicated in mitochondrial fusion/fission and mitophagy, respectively. These results further strengthen the recent evidence indicating that dysregulated mitochondrial network, function, and dynamics are relevant hubs contributing to tumour cell phenotypes, likely by affecting energetic metabolism and redox homeostasis [42,43], and demonstrate, for the first time, that such mitochondrial remodelling might be considered a phenomenon that occurs in MM and could have a role in myelomagenesis. Overall, these data provide the preliminary framework to functionally address the role of mitochondrial quality control systems as novel candidate biomarker for MM onset and progression.

On the basis of previous evidence indicating epigenetic regulation of mitochondrial quality control genes in different pathophysiological contexts [35,37], we next sought to investigate specific epigenetic marks in MM cells, and thus focused on CpG methylation. Since methylation-specific PCR (MSP), semi-quantitative real-time PCR and bisulfite sequencing suffer from poor versatility, limited quantitative resolution and are affected by restricted CpG coverage, we exploited the Sequenom MassARRAY EpiTYPER technology that seems more appropriate for a quantitative assessment for multiple CpG site methylation within candidate genes [44].

In accordance with previous data showing that mitochondrial quality control genes can be regulated by DNA methylation marks in both human and animal models [35,37], our findings indicated peculiar CpG DNA methylation patterns for those genes differentially expressed in MM, mainly in a trend towards hypermethylation. Specifically, *KIF5B*, *MAP1LC3A*, *MARCH5*, *MFN1*, *MFN2*, *MTERF1*, *MTERF3*, *MTIF3*, *POLG2*, *POLRMT*, *RAB32*, *RHOT1*, *TFAM*, and *TFB1M* genes were found hypermethylated in both HMCLs while just *COX10* gene displayed hypomethylation; no statistically significant change in methylation levels was observed in the *COX18*, *FIS1*, *DNM1L*, *MTIF2*, and *TFB2M* genes.

Intriguingly, we noticed that only a few of the genes analysed displayed the expected negative correlation between the mRNA expression pattern and the methylation levels of their associated CpG islands. In fact, a decrease in mean methylation of the relative CpG islands in both HMCLs was associated with increased mRNA levels only for *COX10*, *MAP1LC3*, *MTIF3*, and *RAB32* genes, while CpGs hypomethylation in both MM cell lines was related to higher expression levels of *COX10* mRNA in pathological samples. Whether CpG methylation occurs in genomic regions relevant from a functional point of view, and/or the expression of these genes is regulated by transcription factors sensitive to methylated CpGs, will be assessed in follow-up functional studies.

Of note, given the high heterogeneity of MM cells at genomic and epigenomic level, further studies, enlarging the number of MM samples and including different normal and malignant primary PCs, are mandatory to fully decipher the CpG methylation status of these genes and their functional consequences in the setting of this disease. However, our results are in line with previous work demonstrating heterogeneous expression and functions in HMCLs of genes associated with mitochondrial functions, as those encoding the mitochondrial pore complex, anti-oxidant proteins, and Ca^2+^ channels [33].

In conclusion, the findings presented in this study indicate dysregulated expression of genes related to mitochondrial functions in MM cells, along with peculiar CpG methylation profiles in HMCLs, underscoring a possible involvement of deranged mitochondrial networks in MM pathobiology.

## Figures and Tables

**Table 1:** Mean of DNA methylation values (and standard deviation) of CpG sites located within the analysed genes in HMCLs (MM1s, KMS11) and normal control cells (PBMCs). HMCLs, human myeloma cell lines; FDR: False Discovery Rate.

	MM1s	KMS11	Normal Control Cells	MM1s vs. Normal Control Cells		KMS11 vs. Normal Control Cells	
Gene				*p*-Value	FDR	*p*-Value	FDR
*COX10*	0.353 (0.003)	0.331 (0.009)	0.528 (0.014)	3.57 × 10^−6^	1.67 × 10^−5^	2.91 × 10^−6^	1.36 × 10^−5^
*COX18*	0.054 (0.003)	0.058 (0.006)	0.068 (0.007)	0.134	0.250	0.406	0.421
*DNM1L*	0.071 (0.002)	0.056 (0.002)	0.065 (0.003)	0.796	0.872	0.101	0.129
*FIS1*	0.091 (0.014)	0.104 (0.007)	0.082 (0.009)	0.766	0.872	0.188	0.229
*KIF5B*	0.043 (0.006)	0.056 (0.003)	0.044 (0.003)	0.473	0.698	0.055	0.077
*MAP1LC3A*	0.760 (0.007)	0.836 (0.005)	0.262 (0.009)	8.45 × 10^−16^	7.89 × 10^−15^	6.67 × 10^−14^	9.34 × 10^−13^
*MARCH5*	0.068 (0.003)	0.081 (0.006)	0.057 (0.002)	0.052	0.120	8.09 × 10^−4^	0.002
*MFN1*	0.090 (0.014)	0.105 (0.009)	0.052 (0.003)	0.003	0.008	2.22 × 10^−5^	7.76 × 10^−5^
*MFN2*	0.064 (0.004)	0.075 (0.007)	0.057 (0.004)	0.177	0.292	0.029	0.046
*MTERF*	0.058 (0.009)	0.159 (0.008)	0.043 (0.005)	0.939	0.939	1.24 × 10^−8^	6.93 × 10^−8^
*MTERFD1*	0.091 (0.004)	0.086 (0.009)	0.056 (0.003)	1.29 × 10^−6^	7.24 × 10^−6^	0.001	0.002
*MTIF2*	0.053 (0.01)	0.057 (0.004)	0.039 (0.009)	0.143	0.250	0.213	0.249
*MTIF3*	0.064 (0.008)	0.074 (0.008)	0.049 (0.002)	0.029	0.075	0.002	0.003
*POLG1*	0.492 (0.014)	0.525 (0.003)	0.510 (0.008)	0.133	0.250	0.235	0.254
*POLG2*	0.060 (0.002)	0.070 (0.009)	0.036 (0.003)	4.14 × 10^−5^	1.45 × 10^−4^	9.26 × 10^−4^	0.002
*POLRMT*	0.066 (0.002)	0.060 (0.005)	0.027 (0.005)	9.73 × 10^−6^	3.89 × 10^−5^	0.003	0.005
*RAB32*	0.358 (0.003)	0.596 (0.015)	0.079 (0.006)	3.15 × 10^−19^	8.8 × 10^−18^	5.21 × 10^−14^	9.34 × 10^−13^
*RHOT1*	0.042 (0.003)	0.048 (0.007)	0.037 (0.002)	0.809	0.872	0.082	0.109
*TFAM*	0.065 (0.004)	0.076 (0.003)	0.047 (0.003)	0.894	0.927	1.37x10^−4^	4.27 × 10^−4^
*TFB1M*	0.103 (0.002)	0.168 (0.008)	0.088 (0.006)	0.653	0.831	1.29x10^−5^	5.15 × 10^−0.5^
*TFB2M*	0.090 (0.010)	0.077 (0.009)	0.074 (0.009)	0.556	0.779	0.850	0.850

## Supplementary Materials

The following are available online at https://www.mdpi.com/2077-0383/10/6/1295/s1, Table S1: Association analysis between methylation levels of CpG sites falling within genes involved in mitochondrial quality control observed in HMCLs (MM1s and KMS11) with respect to healthy control subjects (PBMCs).

## References

[1] Brigle K., Rogers B. (2017). Pathobiology and Diagnosis of Multiple Myeloma. Semin. Oncol. Nurs..

[2] Kazandjian D. (2016). Multiple myeloma epidemiology and survival: A unique malignancy. Semin. Oncol..

[3] Korde N., Kristinsson S.Y., Landgren O. (2011). Monoclonal gammopathy of undetermined significance (MGUS) and smoldering multiple myeloma (SMM): Novel biological insights and development of early treatment strategies. Blood.

[4] Landgren O., Kyle R.A., Pfeiffer R.M., Katzmann J.A., Caporaso N.E., Hayes R.B., Dispenzieri A., Kumar S., Clark R.J., Baris D. (2009). Monoclonal gammopathy of undetermined significance (MGUS) consistently precedes multiple myeloma: A prospective study. Blood.

[5] Alagpulinsa D.A., Szalat R.E., Poznansky M.C., Shmookler Reis R.J. (2020). Genomic Instability in Multiple Myeloma. Trends Cancer.

[6] Castaneda O., Baz R. (2019). Multiple Myeloma Genomics—A Concise Review. Acta Med. Acad..

[7] Manier S., Salem K.Z., Park J., Landau D.A., Getz G., Ghobrial I.M. (2017). Genomic complexity of multiple myeloma and its clinical implications. Nat. Rev. Clin. Oncol..

[8] Weaver C.J., Tariman J.D. (2017). Multiple Myeloma Genomics: A Systematic Review. Semin. Oncol. Nurs..

[9] Morelli E., Gulla A., Rocca R., Federico C., Raimondi L., Malvestiti S., Agosti V., Rossi M., Costa G., Giavaresi G. (2020). The Non-Coding RNA Landscape of Plasma Cell Dyscrasias. Cancers.

[10] De Smedt E., Lui H., Maes K., De Veirman K., Menu E., Vanderkerken K., De Bruyne E. (2018). The Epigenome in Multiple Myeloma: Impact on Tumor Cell Plasticity and Drug Response. Front. Oncol..

[11] Amodio N., D’Aquila P., Passarino G., Tassone P., Bellizzi D. (2017). Epigenetic modifications in multiple myeloma: Recent advances on the role of DNA and histone methylation. Expert Opin. Ther. Targets.

[12] Fulciniti M., Amodio N., Cea M., Maiso P., Azab A.K. (2016). Biological Insights into Myeloma and Other B Cell Malignancies. Biomed. Res. Int..

[13] Agirre X., Castellano G., Pascual M., Heath S., Kulis M., Segura V., Bergmann A., Esteve A., Merkel A., Raineri E. (2015). Whole-epigenome analysis in multiple myeloma reveals DNA hypermethylation of B cell-specific enhancers. Genome Res..

[14] Abdi J., Qiu L., Chang H. (2014). Micro-RNAs, New performers in multiple myeloma bone marrow microenvironment. Biomark. Res..

[15] Popovic R., Martinez-Garcia E., Giannopoulou E.G., Zhang Q., Zhang Q., Ezponda T., Shah M.Y., Zheng Y., Will C.M., Small E.C. (2014). Histone methyltransferase MMSET/NSD2 alters EZH2 binding and reprograms the myeloma epigenome through global and focal changes in H3K36 and H3K27 methylation. PLoS Genet..

[16] Walker B.A., Wardell C.P., Chiecchio L., Smith E.M., Boyd K.D., Neri A., Davies F.E., Ross F.M., Morgan G.J. (2011). Aberrant global methylation patterns affect the molecular pathogenesis and prognosis of multiple myeloma. Blood.

[17] Raimondi L., De Luca A., Morelli E., Giavaresi G., Tagliaferri P., Tassone P., Amodio N. (2016). MicroRNAs: Novel Crossroads between Myeloma Cells and the Bone Marrow Microenvironment. BioMed Res. Int..

[18] Pawlyn C., Kaiser M.F., Heuck C., Melchor L., Wardell C.P., Murison A., Chavan S.S., Johnson D.C., Begum D.B., Dahir N.M. (2016). The Spectrum and Clinical Impact of Epigenetic Modifier Mutations in Myeloma. Clin. Cancer Res..

[19] Dasgupta A., Shukla S.K., Gunda V., King R.J., Singh P.K. (2019). Evaluating the Metabolic Alterations in Pancreatic Cancer. Methods Mol. Biol..

[20] Moro L. (2019). Mitochondrial Dysfunction in Aging and Cancer. J. Clin. Med..

[21] Errichiello E., Venesio T. (2017). Mitochondrial DNA variants in colorectal carcinogenesis: Drivers or passengers?. J. Cancer Res. Clin. Oncol..

[22] Yadav N., Chandra D. (2013). Mitochondrial DNA mutations and breast tumorigenesis. Biochim. Biophys. Acta.

[23] Amer W., Toth C., Vassella E., Meinrath J., Koitzsch U., Arens A., Huang J., Eischeid H., Adam A., Buettner R. (2017). Evolution analysis of heterogeneous non-small cell lung carcinoma by ultra-deep sequencing of the mitochondrial genome. Sci. Rep..

[24] Hopkins J.F., Denroche R.E., Aguiar J.A., Notta F., Connor A.A., Wilson J.M., Stein L.D., Gallinger S., Boutros P.C. (2018). Mutations in Mitochondrial DNA From Pancreatic Ductal Adenocarcinomas Associate With Survival Times of Patients and Accumulate as Tumors Progress. Gastroenterology.

[25] Roth K.G., Mambetsariev I., Kulkarni P., Salgia R. (2020). The Mitochondrion as an Emerging Therapeutic Target in Cancer. Trends Mol. Med..

[26] Dai W., Jiang L. (2019). Dysregulated Mitochondrial Dynamics and Metabolism in Obesity, Diabetes, and Cancer. Front. Endocrinol. (Lausanne).

[27] Maycotte P., Marin-Hernandez A., Goyri-Aguirre M., Anaya-Ruiz M., Reyes-Leyva J., Cortes-Hernandez P. (2017). Mitochondrial dynamics and cancer. Tumour Biol..

[28] Srinivasan S., Guha M., Kashina A., Avadhani N.G. (2017). Mitochondrial dysfunction and mitochondrial dynamics-The cancer connection. Biochim. Biophys. Acta Bioenerg..

[29] Vyas S., Zaganjor E., Haigis M.C. (2016). Mitochondria and Cancer. Cell.

[30] Senft D., Ronai Z.A. (2016). Regulators of mitochondrial dynamics in cancer. Curr. Opin. Cell Biol..

[31] Rehman J., Zhang H.J., Toth P.T., Zhang Y., Marsboom G., Hong Z., Salgia R., Husain A.N., Wietholt C., Archer S.L. (2012). Inhibition of mitochondrial fission prevents cell cycle progression in lung cancer. FASEB J.

[32] Mancias J.D., Kimmelman A.C. (2016). Mechanisms of Selective Autophagy in Normal Physiology and Cancer. J. Mol. Biol..

[33] Song I.S., Kim H.K., Lee S.R., Jeong S.H., Kim N., Ko K.S., Rhee B.D., Han J. (2013). Mitochondrial modulation decreases the bortezomib-resistance in multiple myeloma cells. Int. J. Cancer.

[34] Zhan X., Yu W., Franqui-Machin R., Bates M.L., Nadiminti K., Cao H., Amendt B.A., Jethava Y., Frech I., Zhan F. (2017). Alteration of mitochondrial biogenesis promotes disease progression in multiple myeloma. Oncotarget.

[35] Mohammed S.A., Ambrosini S., Luscher T., Paneni F., Costantino S. (2020). Epigenetic Control of Mitochondrial Function in the Vasculature. Front. Cardiovasc. Med..

[36] D’Aquila P., Montesanto A., De Rango F., Guarasci F., Passarino G., Bellizzi D. (2019). Epigenetic signature: Implications for mitochondrial quality control in human aging. Aging (Albany NY).

[37] Mohammad H.P., Barbash O., Creasy C.L. (2019). Targeting epigenetic modifications in cancer therapy: Erasing the roadmap to cancer. Nat. Med..

[38] Uittenbogaard M., Brantner C.A., Chiaramello A. (2018). Epigenetic modifiers promote mitochondrial biogenesis and oxidative metabolism leading to enhanced differentiation of neuroprogenitor cells. Cell Death Dis..

[39] Fulciniti M., Amodio N., Bandi R.L., Cagnetta A., Samur M.K., Acharya C., Prabhala R., D’Aquila P., Bellizzi D., Passarino G. (2016). miR-23b/SP1/c-myc forms a feed-forward loop supporting multiple myeloma cell growth. Blood Cancer J..

[40] Ronchetti D., Agnelli L., Pietrelli A., Todoerti K., Manzoni M., Taiana E., Neri A. (2018). A compendium of long non-coding RNAs transcriptional fingerprint in multiple myeloma. Sci. Rep..

[41] Barfield R.T., Kilaru V., Smith A.K., Conneely K.N. (2012). CpGassoc: An R function for analysis of DNA methylation microarray data. Bioinformatics.

[42] Missiroli S., Genovese I., Perrone M., Vezzani B., Vitto V.A.M., Giorgi C. (2020). The Role of Mitochondria in Inflammation: From Cancer to Neurodegenerative Disorders. J. Clin. Med..

[43] Barbato A., Scandura G., Puglisi F., Cambria D., La Spina E., Palumbo G.A., Lazzarino G., Tibullo D., Di Raimondo F., Giallongo C. (2020). Mitochondrial Bioenergetics at the Onset of Drug Resistance in Hematological Malignancies: An Overview. Front. Oncol..

[44] O’Sullivan E., Goggins M. (2013). DNA methylation analysis in human cancer. Methods Mol. Biol..

