# Supplementary material for: Epigenetic Regulation of Mitochondrial Quality Control Genes in Multiple Myeloma: A Sequenom MassARRAY Pilot Investigation on HMCLs
Source: J Clin Med. 2021 Mar 21;10(6):1295. doi: 10.3390/jcm10061295 (PMC8004002; doi:10.3390/jcm10061295)
Supplement: Supplementary file 1 [file jcm-10-01295-s001.pdf]

Supplementary Table 1: Association analysis between methylation levels of CpG sites falling within genes involved in mitochondrial quality control observed in HMCLs (MM1S and KMS11) with respect to healthy control subjects (PBMCs). FDR: False Discovery Rate

	**KMS11 *vs* normal control cells**			**MM1S *vs* normal control cells**		
**CpG unit**	**F-statistic**	**P-value**	**FDR**	**F-statistic**	**P-value**	**FDR**
COX10_CpG_1	0.00	1.000	1.000	0.86	0.372	0.530
COX10_CpG_2.3	0.15	0.710	0.764	1.79	0.205	0.343
COX10_ CpG _4	7.26	0.020	0.039	2.07	0.176	0.309
COX10_ CpG _6	3.03	0.108	0.173	0.43	0.524	0.671
COX10_ CpG _7.8	3.84	0.074	0.124	3.52	0.085	0.174
COX10_ CpG _10.11	0.94	0.350	0.432	1.15	0.304	0.466
COX10_ CpG _12	1.59	0.236	0.324	0.04	0.848	0.903
COX10_ CpG _13	6.96	0.022	0.042	7.48	0.018	0.049
COX18_ CpG _1	0.77	0.398	0.480	0.34	0.569	0.710
COX18_ CpG _2	2.24	0.161	0.240	5.98	0.031	0.077
COX18_ CpG _4.5.6	30.82	1.26E-04	4.42E-04	12.60	0.004	0.013
COX18_ CpG _7.8	4.55	0.056	0.099	2.37	0.152	0.283
COX18_ CpG _9	15.34	0.002	0.006	21.23	6.03E-04	0.003
COX18_ CpG _10	3.95	0.070	0.118	1.84	0.200	0.340
COX18_ CpG _13.14	0.74	0.406	0.482	2.23	0.161	0.290
COX18_ CpG _15	24.29	3.49E-04	0.001	15.72	0.002	0.007
COX18_ CpG _16.17	9.93	0.008	0.019	5.50	0.037	0.090
COX18_ CpG _22.23	0.74	0.406	0.482	4.37	0.059	0.132
COX18_ CpG _24	1.55	0.237	0.324	0.34	0.569	0.710
DNM1L_ CpG _1.2	0.33	0.578	0.637	0.28	0.605	0.728
DNM1L_ CpG _3.4	8.46	0.016	0.032	43.50	6.11E-05	3.41E-04
DNM1L_ CpG _5	19.20	0.022	0.043	4.80	0.116	0.226
DNM1L_ CpG _7.8	0.35	0.568	0.630	4.17	0.068	0.149
DNM1L_ CpG _9	1.60	0.295	0.389	16.20	0.028	0.070
DNM1L_ CpG _10.11.12.13	0.01	0.940	0.959	0.42	0.529	0.672
DNM1L_ CpG _14.15.16	0.14	0.711	0.764	10.52	0.007	0.022
DNM1L_ CpG _17.18.19	0.84	0.378	0.459	1.44	0.253	0.407
DNM1L_ CpG _20	2.24	0.161	0.240	11.69	0.005	0.016
DNM1L_ CpG _28.29.30	1.45	0.252	0.340	0.77	0.398	0.562
DNM1L_ CpG _34	2.92	0.116	0.182	14.62	0.003	0.010
DNM1L_ CpG _35	0.83	0.380	0.461	0.98	0.342	0.505
FIS1_ CpG _1.2	4.48	0.056	0.098	6.12	0.029	0.074
FIS1_ CpG _4	0.67	0.430	0.507	0.08	0.783	0.864
FIS1_ CpG _5	0.67	0.430	0.507	0.08	0.783	0.864
FIS1_ CpG _6.7	0.82	0.384	0.463	0.30	0.593	0.723
KIF5B_ CpG _1.2	1.41	0.300	0.392	1.90	0.240	0.391
KIF5B_ CpG _3	2.99	0.109	0.174	0.32	0.582	0.717
KIF5B_ CpG _11	3.40	0.090	0.147	4.41	0.057	0.130
KIF5B_ CpG _12.13.14	0.09	0.769	0.813	0.59	0.457	0.623

KIF5B_ CpG _15.16	4.19	0.097	0.214	4.34	0.061	0.136
KIF5B_ CpG _17	1.71	0.215	0.303	0.09	0.774	0.861
KIF5B_ CpG _18	0.18	0.680	0.736	0.06	0.815	0.886
KIF5B_ CpG _19.20.21	9.52	0.009	0.021	3.43	0.089	0.178
KIF5B_ CpG _22.23.24.25.26	3.80	0.075	0.126	5.37	0.039	0.094
KIF5B_ CpG _31.32	0.01	0.923	0.946	0.13	0.727	0.825
KIF5B_ CpG _33	9.84	0.009	0.021	0.29	0.600	0.727
MAP1LC3A_1__ CpG _1	589.68	1.43E-11	1.58E-10	299.61	7.48E-10	7.36E-09
MAP1LC3A_1__ CpG _2.3	935.05	5.42E-12	6.31E-11	637.03	4.35E-11	5.13E-10
MAP1LC3A_1__ CpG _4	1037.14	5.08E-13	7.67E-12	2057.28	8.60E-15	2.22E-13
MAP1LC3A_1__ CpG _5	7.69	0.017	0.034	4.96	0.046	0.107
MAP1LC3A_1__ CpG _6.7	9956.83	6.87E-19	1.22E-16	3781.92	2.26E-16	7.78E-15
MAP1LC3A_1__ CpG _8.9	476.32	2.05E-08	1.39E-07	413.39	3.58E-08	3.02E-07
MAP1LC3A_1__ CpG _10	229.27	3.49E-09	2.64E-08	161.19	2.57E-08	2.26E-07
MAP1LC3A_1__ CpG _11.12	466.50	2.22E-08	1.46E-07	413.39	3.58E-08	3.02E-07
MAP1LC3A_1__ CpG _13.14	1538.88	4.86E-14	1.17E-12	1595.59	3.92E-14	7.35E-13
MAP1LC3A_1__ CpG _15	1068.83	4.25E-13	6.93E-12	557.20	2.00E-11	2.58E-10
MAP1LC3A_1__ CpG _16	597.67	1.32E-11	1.50E-10	299.61	7.48E-10	7.36E-09
MAP1LC3A_1__ CpG _17.18	9517.70	9.00E-19	1.22E-16	13969.34	9.02E-20	1.24E-17
MAP1LC3A_1__ CpG _19.20.21	20038.93	2.74E-19	1.12E-16	6642.27	1.18E-16	4.88E-15
MAP1LC3A_1__ CpG _22.23.24.25	6117.03	1.27E-17	1.30E-15	5655.36	2.03E-17	1.40E-15
MAP1LC3A_1__ CpG _26	5187.82	3.41E-17	2.32E-15	4908.83	4.75E-17	2.45E-15
MAP1LC3A_1__ CpG _27.28	1564.59	4.40E-14	1.12E-12	1595.59	3.92E-14	7.35E-13
MAP1LC3A_1__ CpG _29	2004.03	1.01E-14	3.42E-13	1276.91	1.48E-13	2.44E-12
MAP1LC3A_1__ CpG _30	1818.76	1.79E-14	5.63E-13	1875.64	1.49E-14	3.36E-13
MAP1LC3A_1__ CpG _32	489.68	4.27E-11	3.87E-10	336.34	3.83E-10	4.16E-09
MAP1LC3A_1__ CpG _33.34	3459.77	3.85E-16	1.96E-14	3459.66	3.85E-16	1.16E-14
MAP1LC3A_1__ CpG _35	171.51	1.82E-08	1.26E-07	109.14	2.23E-07	1.74E-06
MAP1LC3A_1__ CpG _36	1663.34	3.06E-14	8.31E-13	1506.88	5.50E-14	9.88E-13
MAP1LC3A_1__ CpG _37	576.83	1.63E-11	1.75E-10	299.61	7.48E-10	7.36E-09
MAP1LC3A_1__ CpG _38	1096.01	3.66E-13	6.22E-12	557.20	2.00E-11	2.58E-10
MAP1LC3A_1__ CpG _39	574.08	1.68E-11	1.75E-10	299.61	7.48E-10	7.36E-09
MAP1LC3A_1__ CpG _40	4557.14	7.41E-17	4.32E-15	532.34	2.61E-11	3.18E-10
MAP1LC3A_1__ CpG _44.45	33.82	8.27E-05	3.01E-04	27.03	2.22E-04	0.001
MAP1LC3A_2_ CpG _6.7	5.48	0.037	0.070	4.42	0.057	0.130
MAP1LC3A_2_ CpG _8.9	29.96	1.42E-04	4.95E-04	24.83	3.18E-04	0.001
MAP1LC3A_2_ CpG _10	29.24	1.58E-04	5.47E-04	7.72	0.017	0.046
MAP1LC3A_2_ CpG _11.12.13	48.68	1.48E-05	6.36E-05	37.88	4.91E-05	2.82E-04
MAP1LC3A_2_ CpG _15	1.79	0.206	0.293	1.78	0.207	0.344
MAP1LC3A_2_ CpG _16.17	7.76	0.016	0.034	0.22	0.647	0.761
MAP1LC3A_2_ CpG _18	17.84	0.001	0.003	0.12	0.740	0.834
MAP1LC3A_2_ CpG _19	0.28	0.607	0.667	0.46	0.511	0.657
MAP1LC3A_2_ CpG _30	100.76	5.54E-04	0.002	0.01	0.945	0.957
MAP1LC3A_2_ CpG _31	12.96	0.009	0.020	6.61	0.037	0.090
MAP1LC3B_ CpG _1	1982.79	8.95E-14	1.66E-12	699.80	2.61E-11	3.18E-10
MAP1LC3B_ CpG _3	2.38	0.157	0.238	1.91	0.200	0.340

MAP1LC3B_ CpG _4.5.6	883.76	1.31E-12	1.91E-11	2.71	0.126	0.242
MAP1LC3B_ CpG _7	183.86	3.28E-08	2.06E-07	47.42	2.63E-05	1.65E-04
MAP1LC3B_ CpG _8	145.01	4.65E-08	2.88E-07	10.20	0.008	0.023
MAP1LC3B_ CpG _9	851.33	1.64E-12	2.23E-11	246.74	2.29E-09	2.10E-08
MAP1LC3B_ CpG _10.11	813.89	2.14E-12	2.81E-11	256.02	1.85E-09	1.74E-08
MAP1LC3B_ CpG _12.13	5263.37	3.13E-17	2.32E-15	1014.80	5.78E-13	9.18E-12
MAP1LC3B_ CpG _14.15.16	2490.67	2.75E-15	1.25E-13	1936.33	1.23E-14	3.00E-13
MAP1LC3B_ CpG _18	183.86	3.28E-08	2.06E-07	35.96	8.97E-05	4.63E-04
MAP1LC3B_ CpG _20.21.22	16.60	0.002	0.006	0.10	0.755	0.846
MAP1LC3B_ CpG _24.25	113.00	1.84E-07	1.04E-06	5.55	0.036	0.089
MAP1LC3B_ CpG _26.27	113.00	1.84E-07	1.04E-06	7.36	0.019	0.051
MAP1LC3B_ CpG _28	44.97	8.80E-05	3.18E-04	32.81	2.84E-04	0.001
MARCH5_1_ CpG_1	20.74	6.62E-04	0.002	0.52	0.486	0.645
MARCH5_1_ CpG _2.3.4	0.35	0.563	0.628	0.86	0.373	0.530
MARCH5_1_ CpG _5	0.43	0.523	0.590	0.31	0.590	0.722
MARCH5_1_ CpG _6.7	1.59	0.231	0.318	0.29	0.599	0.727
MARCH5_1_ CpG _9.10.11	12.20	0.004	0.011	2.52	0.138	0.262
MARCH5_1_ CpG _12	5.71	0.062	0.106	0.54	0.502	0.652
MARCH5_2_ CpG _1.2	2.27	0.175	0.254	1.46	0.266	0.423
MARCH5_2_ CpG _6.7	10.72	0.007	0.016	1.16	0.303	0.464
MARCH5_2_ CpG _9.10	2.16	0.172	0.250	2.16	0.172	0.307
MARCH5_2_ CpG _11.12	0.01	0.913	0.939	0.07	0.797	0.875
MARCH5_2_ CpG _13.14.15.16	9.20	0.010	0.023	0.10	0.756	0.846
MARCH5_2_ CpG _17	1.20	0.299	0.392	1.38	0.270	0.426
MARCH5_2_ CpG _18.19	21.91	5.32E-04	0.002	0.70	0.420	0.584
MARCH5_2_ CpG _22.23.24	0.03	0.864	0.897	1.36	0.267	0.424
MARCH5_2_ CpG _25.26.27	4.34	0.059	0.102	0.65	0.437	0.601
MARCH5_2_ CpG _33	0.76	0.402	0.480	18.31	0.001	0.004
MARCH5_2_ CpG _34.35.36	2.22	0.162	0.240	1.31	0.274	0.429
MARCH5_2_ CpG _38	9.13	0.011	0.023	2.27	0.158	0.287
MARCH5_2_ CpG _39.40.41.42.43	0.21	0.658	0.718	1.06	0.323	0.484
MARCH5_2_ CpG _44	37.61	5.08E-05	1.97E-04	49.30	1.39E-05	9.42E-05
MARCH5_2_ CpG _45.46	2.75	0.123	0.192	0.19	0.667	0.781
MARCH5_3_ CpG v_1	36.01	6.21E-05	2.37E-04	17.80	0.001	0.005
MARCH5_3_ CpG _7	26.21	2.53E-04	8.55E-04	3.11	0.103	0.203
MFN1_ CpG _1.2	0.36	0.557	0.623	0.21	0.653	0.766
MFN1_ CpG _6	9.84	0.014	0.029	10.15	0.032	0.047
MFN1_ CpG _9.10	38.11	4.77E-05	1.89E-04	6.48	0.027	0.070
MFN1_ CpG _11	68.58	4.19E-04	0.001	525.00	2.94E-06	2.13E-05
MFN1_ CpG _17.18	3.44	0.088	0.146	0.47	0.507	0.655
MFN1_ CpG _3.4.5	10.49	0.007	0.017	0.51	0.489	0.647
MFN2_ CpG _1	1.15	0.332	0.413	0.09	0.782	0.864
MFN2_ CpG _5.6.7.8	13.35	0.003	0.009	3.45	0.088	0.178
MFN2_ CpG _9.10.11	1.10	0.316	0.406	0.43	0.527	0.671
MFN2_ CpG _12.13	23.79	3.80E-04	0.001	1.35	0.268	0.425
MFN2_ CpG _14.15.16	2.19	0.21	0.384	2.06	0.189	0.326

MFN2_ CpG _17.18	6.74	0.023	0.045	0.67	0.429	0.592
MFN2_ CpG _19.20	0.37	0.552	0.619	0.02	0.878	0.920
MFN2_ CpG _21	1.72	0.222	0.310	0.50	0.504	0.653
MFN2_ CpG _22.23	2.35	0.164	0.241	0.37	0.562	0.706
MFN2_ CpG _24	13.17	0.003	0.009	8.80	0.012	0.034
MTERF1_ CpG _1.2	113.36	1.81E-07	1.04E-06	12.25	0.004	0.014
MTERF1_ CpG _3.4	12.60	0.004	0.010	2.45	0.143	0.269
MTERF1_ CpG _5	2.71	0.126	0.196	0.13	0.721	0.822
MTERF1_ CpG _6	763.23	3.12E-12	3.98E-11	9.02	0.011	0.032
MTERF1_ CpG _7	1271.71	1.51E-13	2.68E-12	1.96	0.187	0.324
MTERFD1_1_ CpG _1.2	34.27	7.79E-05	2.92E-04	16.22	0.002	0.006
MTERFD1_1_ CpG _3.4.5.6	2.41	0.147	0.223	4.30	0.060	0.134
MTERFD1_1_ CpG _7	31.57	1.13E-04	4.00E-04	30.19	1.37E-04	6.93E-04
MTERFD1_1_ CpG _8.9	0.65	0.441	0.516	0.04	0.841	0.902
MTERFD1_1_ CpG _10	7.38	0.019	0.038	14.43	0.003	0.009
MTERFD1_1_ CpG _11	0.63	0.444	0.518	4.67	0.052	0.119
MTERFD1_1_ CpG _12	25.26	2.96E-04	9.75E-04	41.24	3.30E-05	2.03E-04
MTERFD1_1_ CpG _13	1.05	0.325	0.408	0.02	0.901	0.935
MTERFD1_1_ CpG _14	19.92	7.75E-04	0.002	58.59	5.89E-06	4.12E-05
MTERFD1_1_ CpG _16.17	54.68	8.33E-06	3.78E-05	85.94	8.07E-07	6.06E-06
MTERFD1_1_ CpG _24.25	4.60	0.053	0.095	0.04	0.847	0.903
MTERFD1_1_ CpG _26	24.51	3.36E-04	0.001	115.79	1.61E-07	1.28E-06
MTERFD1_1_ CpG _27	1.16	0.317	0.406	1.12	0.325	0.485
MTERFD1_1_ CpG _28.29.30	4.21	0.063	0.106	0.03	0.867	0.916
MTERFD1_1_ CpG _31.32.33.34	5.87	0.032	0.061	19.91	7.77E-04	0.003
MTERFD1_1_ CpG _35	1.05	0.325	0.408	0.11	0.741	0.834
MTERFD1_1_ CpG _36	36.68	5.70E-05	2.20E-04	43.50	2.55E-05	1.62E-04
MTERFD1_1_ CpG _37.38	1.96	0.187	0.268	0.49	0.499	0.652
MTERFD1_1_ CpG _39	0.00	0.960	0.974	0.15	0.707	0.813
MTERFD1_1_ CpG _40	1.29	0.278	0.369	4.87	0.047	0.110
MTERFD1_1_ CpG _41	182.43	1.28E-08	9.17E-08	277.15	1.17E-09	1.13E-08
MTERFD1_1_ CpG _42.43	1.78	0.209	0.296	3.67	0.082	0.170
MTERFD1_1_ CpG _44	9.83	0.009	0.020	0.01	0.916	0.939
MTIF2_ CpG _1	0.44	0.519	0.589	0.15	0.707	0.813
MTIF2_ CpG _3.4	0.56	0.467	0.540	25.54	2.83E-04	0.001
MTIF2_ CpG _5	0.05	0.824	0.862	0.46	0.523	0.671
MTIF2_ CpG _7	1.26	0.299	0.392	1.96	0.204	0.343
MTIF2_ CpG _8	2.63	0.131	0.201	3.32	0.093	0.186
MTIF2_ CpG _9.10	2.48	0.141	0.216	0.97	0.345	0.507
MTIF2_ CpG _11	139.76	5.71E-08	3.48E-07	21.88	5.34E-04	0.002
MTIF2_ CpG _12	0.57	0.465	0.539	0.33	0.574	0.714
MTIF2_ CpG _13.14	8.10	0.015	0.031	0.04	0.847	0.903
MTIF2_ CpG _20	1.05	0.325	0.408	0.40	0.537	0.680
MTIF2_ CpG _28	14.89	0.002	0.006	9.55	0.009	0.028
MTIF3_1_ CpG _1	9.74	0.009	0.020	0.87	0.369	0.530
MTIF3_1_ CpG _2	21.10	6.18E-04	0.002	25.00	3.09E-04	0.001

MTIF3_1_CpG_3	12.04	0.005	0.011	1.80	0.204	0.343
MTIF3_1_CpG_4	21.09	6.19E-04	0.002	48.00	1.59E-05	1.06E-04
MTIF3_1_CpG_5.6.7	14.73	0.002	0.006	25.29	2.95E-04	0.001
MTIF3_1_CpG_8	0.61	0.451	0.524	2.60	0.133	0.253
MTIF3_1_CpG_9	1.09	0.317	0.406	0.01	0.908	0.936
MTIF3_1_CpG_10.11	21.80	5.42E-04	0.002	5.42	0.038	0.092
MTIF3_1_CpG_12	9.74	0.009	0.020	1.03	0.330	0.488
MTIF3_1_CpG_13	1.09	0.317	0.406	0.49	0.496	0.652
MTIF3_1_CpG_17.18.19.20	80.63	1.13E-06	5.77E-06	150.86	3.73E-08	3.08E-07
MTIF3_1_CpG_22.23	7.94	0.016	0.032	8.57	0.013	0.036
MTIF3_1_CpG_24	20.09	7.49E-04	0.002	23.25	4.17E-04	0.002
MTIF3_3_CpG_1.2	17.12	0.001	0.004	0.03	0.860	0.911
MTIF3_3_CpG_3	3.63	0.081	0.134	4.21	0.065	0.143
MTIF3_3_CpG_4.5	1.13	0.309	0.400	2.05	0.180	0.313
MTIF3_3_CpG_6.7	12.50	0.004	0.010	1.28	0.280	0.437
MTIF3_3_CpG_8	2.22	0.162	0.240	3.64	0.080	0.169
MTIF3_3_CpG_9	1.02	0.331	0.413	0.86	0.371	0.530
MTIF3_3_CpG_10	1.02	0.335	0.415	3.73	0.082	0.170
MTIF3_3_CpG_11	35.20	6.89E-05	2.60E-04	0.60	0.452	0.619
MTIF3_3_CpG_12	4.33	0.060	0.102	0.31	0.588	0.720
MTIF3_3_CpG_13	61.57	4.58E-06	2.22E-05	5.09	0.044	0.103
MTIF3_3_CpG_14	0.13	0.725	0.772	0.87	0.372	0.530
MTIF3_3_CpG_15.16	11.64	0.005	0.013	19.95	7.71E-04	0.003
MTIF3_3_CpG_17	10.46	0.007	0.017	3.00	0.109	0.213
POLG_1_CpG_1	1.17	0.301	0.392	0.00	0.977	0.981
POLG_1_CpG_2	0.00	0.946	0.963	0.01	0.930	0.944
POLG_1_CpG_10	4.43	0.057	0.100	1.12	0.310	0.471
POLG_1_Cpg_11.12.13	0.48	0.501	0.574	0.00	0.952	0.961
POLG_1_CpG_14.15.16	0.05	0.828	0.862	1.64	0.224	0.367
POLG_1_CpG_17	0.44	0.522	0.590	0.01	0.926	0.944
POLG_1_CpG_18	0.43	0.523	0.590	0.02	0.904	0.936
POLG_1_CpG_19	5.73	0.034	0.064	2.29	0.156	0.287
POLG_1_CpG_20	0.63	0.442	0.516	1.09	0.316	0.479
POLG_1_CpG_21.22	2.67	0.128	0.199	0.06	0.819	0.886
POLG_1_CpG_23	3.39	0.090	0.147	1.21	0.292	0.454
POLG_1_CpG_3	4.55	0.054	0.096	3.66	0.080	0.169
POLG_1_CpG_4	1.97	0.186	0.267	1.81	0.203	0.343
POLG_1_CpG_5	0.20	0.663	0.721	10.31	0.007	0.023
POLG_1_ CpG_6.7	0.05	0.828	0.862	0.52	0.483	0.645
POLG_1_CpG_8	1.48	0.248	0.337	2.08	0.175	0.308
POLG_1_CpG_9	1.47	0.249	0.338	13.26	0.003	0.012
POLG_2_CpG_1.2.3.4	11.77	0.005	0.012	40.84	3.45E-05	2.10E-04
POLG_2_CpG_6.7.8.9	9.31	0.010	0.022	19.93	7.73E-04	0.003
POLG_2_CpG_10.11	0.03	0.876	0.907	6.83	0.023	0.060
POLG_2_CpG_12	0.01	0.940	0.959	7.72	0.017	0.046
POLG_2_CpG_16	1.77	0.208	0.295	1.20	0.295	0.457

POLG_2_CpG_23	8.15	0.015	0.031	0.24	0.635	0.751
POLG_2_CpG_24	12.32	0.004	0.011	21.19	6.07E-04	0.003
POLG_2_CpG_29	2.25	0.160	0.240	5.05	0.044	0.104
POLG_2_CpG_30	6.65	0.024	0.047	9.09	0.011	0.032
POLG_2_CpG_31.32	62.71	4.17E-06	2.08E-05	16.99	0.001	0.005
POLG_2_CpG_33	20.96	7.92E-04	0.002	20.13	0.001	0.005
POLG_2_CpG_34	0.00	0.989	0.992	0.56	0.471	0.635
POLG_2_CpG_35.36	16.02	0.001	0.009	17.55	0.004	0.014
POLG_2_CpG_37	51.12	1.16E-05	5.17E-05	37.93	4.88E-05	2.82E-04
POLG_2_CpG_38.39.40.41	26.11	2.57E-04	8.61E-04	13.26	0.003	0.012
POLG_2_CpG_43.44.45	4.41	0.057	0.100	16.09	0.002	0.006
POLG2_CpG_1	10.02	0.008	0.019	0.70	0.418	0.584
POLG2_CpG_3	1.46	0.258	0.345	4.25	0.069	0.150
POLG2_CpG_4	8.52	0.013	0.027	7.80	0.016	0.045
POLG2_CpG_6	2.05	0.178	0.256	2.77	0.122	0.235
POLG2_CpG_7	10.02	0.008	0.019	1.19	0.296	0.457
POLG2_CpG_8.9	0.01	0.908	0.936	0.33	0.578	0.715
POLG2_CpG_10	6.32	0.029	0.055	0.31	0.587	0.720
POLG2_CpG_12.13.14	19.50	8.42E-04	0.002	20.40	7.06E-04	0.003
POLG2_CpG_15	9.58	0.009	0.021	0.00	0.981	0.984
POLG2_Cpg_16	1.46	0.258	0.345	4.82	0.056	0.128
POLG2_CpG_17.18.19	2.97	0.110	0.175	2.10	0.173	0.308
POLG2_CpG_20.21	20.49	6.94E-04	0.002	90.56	6.10E-07	4.67E-06
POLG2_CpG_22	7.75	0.017	0.034	12.82	0.004	0.013
POLG2_Cpg_23.24.25	2.56	0.135	0.208	0.03	0.870	0.917
POLRMT_2_CpG_5	0.05	0.825	0.862	0.28	0.608	0.728
POLRMT_2_CpG_10	131.61	7.97E-08	4.71E-07	131.61	7.97E-08	6.45E-07
RAB32_1_CpG_4.5.6	0.58	0.568	0.723	0.49	0.502	0.652
RAB32_1_CpG_7.8	186.26	3.07E-08	1.99E-07	0.16	0.700	0.812
RAB32_1_CpG_9	639.64	1.14E-09	9.09E-09	3.76	0.084	0.174
RAB32_1_CpG_10	326.29	2.22E-08	1.46E-07	2.38	0.157	0.287
RAB32_1_CpG_11.12.13	0.97	0.401	0.667	0.86	0.372	0.530
RAB32_1_CpG_14.15	59.90	5.27E-06	2.44E-05	0.25	0.628	0.747
RAB32_1_CpG_24	110.49	2.08E-07	1.15E-06	2.32	0.154	0.285
RAB32_1_CpG_25.26	52.20	1.05E-05	4.71E-05	0.70	0.420	0.584
RAB32_1_CpG_27.28.29.30	512.12	3.28E-11	3.11E-10	1.67	0.220	0.362
RAB32_1_CpG_31.32	188.14	1.08E-08	7.84E-08	6.54	0.025	0.066
RAB32_1_CpG_40.41	10.19	0.019	0.038	0.06	0.819	0.886
RAB32_1_CpG_42	231.61	3.29E-09	2.53E-08	0.39	0.542	0.684
RAB32_1_CpG_43	91.74	5.12E-06	2.40E-05	10.08	0.011	0.033
RAB32_1_CpG_45.46.47	418.35	4.20E-10	3.43E-09	32.53	1.38E-04	6.93E-04
RAB32_1_CpG_48	91.74	5.12E-06	2.40E-05	12.24	0.007	0.021
RAB32_1_CpG_49	245.64	2.35E-09	1.84E-08	18.29	0.001	0.004
RAB32_1_CpG_50.51	551.45	2.12E-11	2.11E-10	34.34	7.72E-05	4.14E-04
RAB32_1_CpG_52	192.18	9.54E-09	7.08E-08	0.88	0.368	0.530
RAB32_1_Cpg_57.58	1045.10	4.85E-13	7.61E-12	6.80	0.023	0.060

RAB32_1_CpG_59.60	33.84	8.25E-05	3.01E-04	1.43	0.255	0.408
RAB32_2_CpG_1.2	85.67	8.20E-07	4.24E-06	68.11	2.73E-06	2.01E-05
RAB32_2_CpG_3	2787.76	1.58E-12	2.22E-11	1611.62	1.84E-11	2.53E-10
RAB32_2_CpG_4	25.94	6.52E-04	0.002	22.14	0.001	0.004
RAB32_2_CpG_7	818.43	1.64E-08	1.15E-07	781.23	1.93E-08	1.73E-07
RAB32_2_CpG_8	3860.55	7.35E-11	6.52E-10	2801.42	2.25E-10	2.51E-09
RAB32_2_CpG_10.11	3881.90	2.76E-14	8.04E-13	2046.12	6.71E-13	1.03E-11
RAB32_2_CpG_12.13.14.15	3781.77	7.89E-11	6.85E-10	3339.41	1.22E-10	1.40E-09
RAB32_2_CpG_19	2396.90	3.45E-15	1.41E-13	1864.86	1.55E-14	3.36E-13
RHOT1_CpG_1.2.3.4	2.22	0.162	0.240	0.55	0.473	0.636
RHOT1_CpG_5.6	6.07	0.030	0.057	0.24	0.634	0.751
RHOT1_CpG_7.8	0.15	0.710	0.764	1.51	0.242	0.394
RHOT1_CpG_9.10	4.54	0.055	0.096	7.12	0.020	0.055
RHOT1_CpG_11	0.08	0.787	0.827	2.14	0.174	0.308
RHOT1_Cpg_12	7.01	0.021	0.042	0.78	0.395	0.558
RHOT1_CpG_13	31.75	1.10E-04	3.94E-04	2.41	0.147	0.274
TFAM_CpG_2	4.92	0.047	0.085	0.00	1.000	1.000
TFAM_CpG_3.4	1.63	0.226	0.313	1.47	0.249	0.401
TFAM_CpG_5	4.60	0.053	0.095	7.32	0.019	0.051
TFAM_CpG_6.7	60.96	4.82E-06	2.31E-05	15.93	0.002	0.007
TFAM_CpG_8.9.10.11	4.54	0.055	0.096	3.67	0.079	0.169
TFAM_CpG_12	105.45	2.69E-07	1.46E-06	61.22	4.71E-06	3.36E-05
TFAM_CpG_13.14.15	22.69	4.61E-04	0.001	3.92	0.071	0.152
TFB1M_CpG_1	16.97	0.001	0.004	5.04	0.044	0.104
TFB1M_CpG_2	0.45	0.513	0.586	35.02	7.05E-05	3.88E-04
TFB1M_CpG_3	111.80	1.95E-07	1.09E-06	16.53	0.002	0.006
TFB1M_CpG_5	12.95	0.004	0.009	0.05	0.825	0.887
TFB1M_CpG_6	92.28	5.52E-07	2.89E-06	21.87	5.36E-04	0.002
TFB1M_CpG_7.8	16.51	0.002	0.004	5.73	0.034	0.084
TFB1M_CpG_9.10.11	61.93	4.45E-06	2.19E-05	45.79	2.00E-05	1.31E-04
TFB1M_CpG_12.13	4.41	0.058	0.100	0.88	0.366	0.530
TFB1M_CpG_14	1.71	0.216	0.303	9.24	0.010	0.030
TFB1M_CpG_15.16	19.25	8.85E-04	0.003	0.06	0.807	0.880
TFB1M_CpG_17	5.46	0.038	0.070	0.05	0.825	0.887
TFB1M_CpG_19.20	14.91	0.002	0.006	3.43	0.089	0.178
TFB1M_CpG_21.22	3.08	0.105	0.169	0.02	0.893	0.929
TFB1M_CpG_23	14.69	0.002	0.006	0.02	0.883	0.924
TFB1M_CpG_24	1.71	0.216	0.303	10.21	0.008	0.023
TFB1M_CpG_25.26	55.51	7.73E-06	3.54E-05	11.21	0.006	0.018
TFB1M_CpG_30	0.21	0.715	0.810	0.16	0.695	0.809
TFB1M_CpG_31	8.52	0.013	0.027	0.11	0.741	0.834
TFB2M_CpG_1	44.81	2.22E-05	9.32E-05	36.91	5.54E-05	3.13E-04
TFB2M_CpG_3	4.14	0.076	0.127	2.40	0.160	0.290
TFB2M_CpG_4.5	27.20	2.16E-04	7.35E-04	55.69	7.61E-06	5.24E-05
TFB2M_CpG_6	1.08	0.320	0.406	0.01	0.920	0.940
TFB2M_CpG_7	43.76	2.48E-05	1.01E-04	8.55	0.013	0.036

TFB2M_CpG_8	0.00	0.975	0.983	12.11	0.005	0.016
TFB2M_CpG_9	1.08	0.320	0.406	0.07	0.801	0.875
TFB2M_CpG_13.14	14.21	0.003	0.007	17.64	0.001	0.005
TFB2M_CpG_15	3.42	0.089	0.146	0.48	0.501	0.652
TFB2M_CpG_18.19	22.08	5.16E-04	0.002	4.03	0.068	0.148
TFB2M_CpG_20	1.69	0.218	0.304	6.52	0.025	0.066
TFB2M_CpG_24	3.42	0.089	0.146	0.33	0.577	0.715
TFB2M_CpG_30	0.14	0.718	0.766	6.33	0.027	0.070
TFB2M_CpG_31.32	0.95	0.348	0.431	1.32	0.273	0.429
TFB2M_CpG_33	2.22	0.162	0.240	4.38	0.058	0.132
TFB2M_CpG_34.35	1.38	0.263	0.350	5.11	0.043	0.103
TFB2M_CpG_36	1.08	0.320	0.406	0.07	0.801	0.875
TFB2M_CpG_37	0.00	0.975	0.983	25.65	3.64E-04	0.002
TFB2M_CpG_38	1.43	0.256	0.344	2.29	0.156	0.287
TFB2M_CpG_39	43.76	2.48E-05	1.01E-04	43.76	2.48E-05	1.60E-04
TFB2M_CpG_40.41.42	0.00	0.981	0.986	1.06	0.324	0.484
TFB2M_CpG_43.44	14.21	0.003	0.007	3.61	0.082	0.170
